# A Computational Study on the Hydrodynamics of Bio-Inspired Quadrupedal Paddling

**DOI:** 10.3390/biomimetics10030148

**Published:** 2025-02-27

**Authors:** Yihan Wang, Yumeng Cai, Bin Xie, Chi Zhu, Yunquan Li, Ye Chen

**Affiliations:** 1 Shien-Ming Wu School of Intelligent Engineering, South China University of Technology, Guangzhou 510641, China; wiyihanwang@mail.scut.edu.cn (Y.W.); wicaiyumeng@mail.scut.edu.cn (Y.C.); xieb7160@gmail.com (B.X.); 2 Department of Mechanics and Engineering Science, Peking University, Beijing 100871, China; chi.zhu@pku.edu.cn

**Keywords:** quadrupedal paddling, thrust generation, immersed-boundary method

## Abstract

Due to its exceptional terrain mobility, quadrupedal locomotion has been used in the design of many amphibious robots for broad applications including resource exploration, disaster rescue, and reconnaissance. In this work, swimming of a quadrupedal paddling model is considered, and the effects of the legs’ initial swing angle, swing amplitude, and power phase duration are numerically investigated through three paddling gaits, namely, the trotting gait, the diagonal, and the lateral sequence gaits. Three different modes for drag-based thrust generation, the “Trotting Mode”, the “Hindering Mode”, and the “Separate Mode”, are identified. In the “Trotting Mode”, each pair of diagonal legs contributes equally and alternately to the thrust within the paddling cycle, and its contribution is impaired by the other pair of diagonal legs. In the “Hindering Mode”, the thrust contribution of an individual leg is significantly undermined by the drag resulting from the preceding leg leaving its current power phase and entering the following recovery phase. In the “Separate Mode”, the four legs independently contribute to the total thrust by forming a compact four-peak waveform equally distributed within one paddling cycle. At a given swing amplitude, the leg configuration at peak thrust moment is identical, regardless of initial swing angle and power phase ratio. Meanwhile, a forward-tilted leg configuration with flatter upper- and lower-limb alignment at peak thrust moment consistently indicates a lower thrust generation. Hydrodynamic moments in the diagonal and lateral sequence gaits are much larger than those in the trotting gait. In addition, enhanced thrust is typically accompanied by larger hydrodynamic moments and a higher energy expenditure.

## 1. Introduction

During the past two decades, various types of amphibious robot have been developed with the aim of quickly transitioning between land and water for broad applications including resource exploration, disaster rescue, and reconnaissance [1]. Normally, locomotion on land and locomotion in water are achieved by different mechanisms for amphibious robots. For land locomotion, legged robots have long captured the interest of researchers because of their outstanding mobility and versatility on rough and transitional zones such as sandy and muddy terrains [2,3,4,5,6,7].

A variety of bio-inspired propulsion mechanisms have been employed in water with inspiration drawn from nature. Body undulations, fish fin-like propulsors, and paddling legs are three common methods of bio-inspired waterborne propulsion for amphibious robots. For example, the snake-like robot ACM can propel itself through body undulation or spiral spinning with a “HELIX” mode at a speed of 0.4 m/s [8,9]. A similar snake-like Multi-Link Mobile Robot MLMR II is proposed with forward and lateral turning ability underwater [10]. A salamander robot can reproduce an overall natural-body-like undulation and swim at velocities ranging from 0.07 to 0.12 m/s [11]. In addition to body undulation, bio-inspired fish fins have also been used as propulsors by amphibious robots for efficient underwater locomotion. AmphiRobot-II uses a caudal fin to perform a BCF-type carangiform swimming with a forward speed of up to 45 cm/s [2]. A pair of ribbon fins similar to those of sting rays is manufactured and the maximum MPF forward swimming speed is about 0.31 m/s at an undulating frequency of 2 Hz [12]. Such bio-locomotion using either body undulation or fin flapping relies on the generation of lift-based thrust. In contrast, drag-based thrust represents another mechanism for propulsion, which is usually deployed by terrestrial and semi-aquatic mammals with paddling appendages [13]. Although propulsion through body undulation and fin flapping is known to be more efficient in water, their terrestrial movements capability is very limited and can hardly be compared with legged locomotion [13,14]. Therefore, legged robots are suitable for amphibious applications despite their relatively low swimming efficiency. Recently, learning-based methods have been developed for gait pattern learning and energy efficiency improvement in complex environments [15,16,17,18,19,20]. However, the interaction between legged robots and fluid medium during the learning process remains unexplored. Other approaches, e.g., a combination of wheels for land movement and fins for swimming, may lead to a much more complicated and heavier amphibious robot design that may not be desirable.

Quite a few legged amphibious robots have been developed in the past. For example, a six-legged crab-like robot ALUV is reported to be capable of walking underwater for mine hunting in the surf zone [4]. A cockroach-inspired robot RHex was first built between 1999 and 2004, based on which the subsequent AQUA platform is further developed with underwater mobility [6,21]. AmphiRobot-I uses a transformable fin-leg module and obtains a moving speed of about 0.5 m/s on land and in water [22]. A bio-inspired quadruped amphibious robot with agile legs based on the five-bar mechanism is also constructed with a successful walking and swimming mobility [23]. With the assistance of the computational fluid dynamics (CFD) technique, one is able to study the hydrodynamics and swimming efficiency of underwater locomotion. In this field, body undulation and fin flapping have been extensively researched [24,25,26,27,28,29].

Unfortunately, to date the study of hydrodynamics for the quadrupedal paddling using the CFD technique is scarce. Among the limited existing studies, the force analysis of quadrupedal hydrodynamics depends on the drag equation and an assumption of the drag coefficient $C_{d}$, and the complex three-dimensional geometrical shape of the legs and the interaction between different limbs are ignored [14,27,30,31]. Such simplified force models are easy to use but may lead to significant errors in practice. Relevant CFD research on leg paddling include computational studies on crawfish, beaver-, and turtle-like robots [31,32,33]. Besides, the synchronous and alternate paddling of the two beaver-like bendable webbed feet is reconstructed and the hydrodynamic forces are investigated through both theoretical analysis and CFD simulations. Table 1 is summarized for previous relevant studies on leg paddling using CFD techniques. However, the models in these studies are distinct from the quadrupedal paddling of mammals, which deserves a further in-depth study since a detailed discussion on paddling hydrodynamics and many other factors, such as paddling kinematics and gait sequences, has not been explored yet. Although a qualitative understanding of the drag-based thrust generation caused by paddling legs may be straightforward (e.g., faster backswing of a paddling leg leads to stronger reaction force by surrounding fluid), a detailed quantitative study is necessary to better understand a few important questions: (I) How propulsion thrust is affected by paddling kinematic parameters including initial swing angle, swing amplitude and power phase ratio? (II) How is the propulsion thrust influenced by different gait sequences? (III) How are the hydrodynamic moments and energetics modulated by paddling kinematics and gait sequences? Motivated by the above fact, it is our novelty to solve the three-dimensional flow field and investigate these questions by performing a series of numerical simulations with the aid of a previously developed parallel CFD solver based on an immersed boundary method.

In this work, we first investigate the paddling-induced thrust of a single leg with initial swing angle $\alpha_{0}\in \left[60^{\circ },70^{\circ },80^{\circ }\right]$ and swing amplitude $\beta_{0}\in \left[20^{\circ },30^{\circ },40^{\circ }\right]$ for three fixed power phase ratios DR∈[50%,33%,25%]. Next, we examine the effect of the gait sequence on the quadrupedal paddling thrust generation in the same parameter space of $\alpha_{0}$ and $\beta_{0}$. Three different mechanisms for thrust generation are categorized and analyzed, and the corresponding hydrodynamic moments and energetics are also discussed.

## 2. Model Description and the Numerical Approach

**Figure 1:**
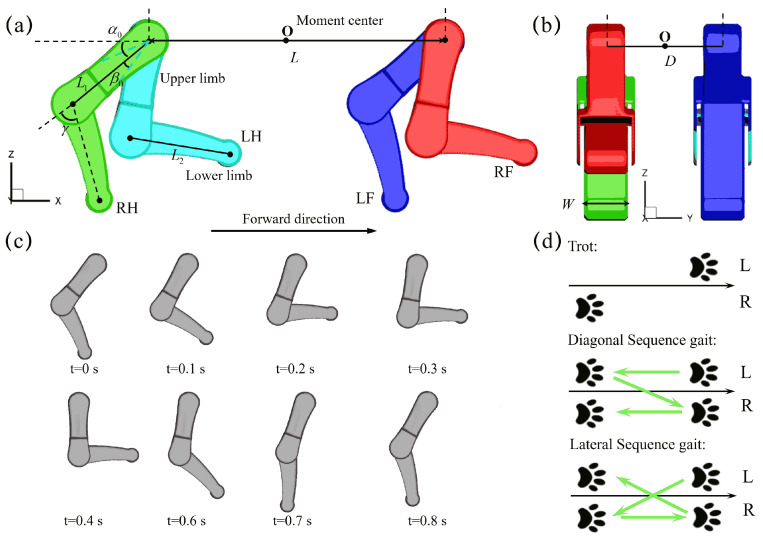
The geometry of the quadrupedal paddling model, (**a**) side view and (**b**) front view. (**c**) Reconstructed paddling sequence of both upper and lower limbs of a single leg under $\alpha_{0}=70^{\circ }$ and $\beta_{0}=20^{\circ }$. (**d**) The three distinct quadrupedal paddling gaits of trotting, the diagonal and lateral sequence gaits considered in this work.

The three-dimensional computational model of quadrupedal paddling used in this work is constructed based on an actual quadrupedal robot and is illustrated in Figure 1a,b. The left-fore leg (LF), left-hind leg (LH), right-fore leg (RF), and right-hind leg (RH) are immersed in an incompressible viscous fluid and perform coordinated paddling motion. The lengths of the upper and lower limbs for each leg, $L_{1}$ and $L_{2}$, are 10.6 and 12.0 cm, respectively. The width *W* of the limb is 4.65 cm. The distance *L* between the fore and hind legs is 32 cm, while the width *D* between the left and right legs is 12.4 cm. These dimensions are taken from an actual robot. A coordinate system is chosen such that its origin O lies in the middle of the upper limb pivots of the four legs. The influence of three major kinematic parameters, the initial swing angle $\alpha_{0}$, the swing amplitude $\beta_{0}$, and the power phase ratio DR are illustrated in the figure. The following values of the parameters are chosen: $\alpha_{0}\in \left[60^{\circ },70^{\circ },80^{\circ }\right]$, $\beta_{0}\in \left[20^{\circ },30^{\circ },40^{\circ }\right]$, and DR∈[25%,33%,50%]. Considering the constant stepping speed of the step motor used in the experimental study in which the duration of the power phase is fixed, DR=50%, 33% and 25% correspond proportionally to a paddling cycle of T=0.8 s, 1.2 s, and 1.6 s, respectively. The prescribed cyclic paddling motion of upper and lower limbs are defined by position angles α(t) and γ(t) (relative to its upper limb), and their transient variations are obtained from the video of the swimming dog provided by [34]. The average lower limb tip velocity $\bar{U}$ within the power phase under DR=50% is 0.64, 0.49 and 0.42 m/s for $\beta_{0}=20^{\circ }$, $30^{\circ }$ and $40^{\circ }$, respectively. This velocity increases further to 0.96, 0.73, 0.60 for DR=33% and 1.34, 1.03, 0.84 m/s for DR=25%, respectively, for $\beta_{0}=20^{\circ }$, $30^{\circ }$ and $40^{\circ }$. Figure 1c presents the kinematic sequence of a single paddling leg under $\alpha_{0}=70^{\circ }$ and $\beta_{0}=20^{\circ }$. In addition to the three paddling kinematic parameters mentioned above, three distinct coordinated leg movements, trotting, diagonal sequence gait, and lateral sequence gait, are also included in this study (Figure 1d). We also point out that when designing an amphibious robotic dog for experimental validation, several key factors must be considered, including actuation, structural stability, waterproofing, and control. The selection of actuators plays a crucial role in swimming performance, and waterproof servos with sufficient torque, such as GXservo-X50 (50 kg·cm), can provide the necessary power for paddling. Additionally, ensuring a proper relationship between the center of buoyancy (CB) and the center of gravity (CG) is essential for maintaining stability in water. Inspired by biological quadrupeds, the CB should be positioned above the CG, reducing the risk of rolling and improving maneuverability. A robust control system can be used to coordinate leg movements, while a wireless remote control allows for flexible gait adjustments. Furthermore, waterproofing measures—including sealed body joints, waterproof adhesives, and protective covers for electronic ports—are essential to prevent water ingress and ensure long-term operation in aquatic environments.

**Figure A1:**
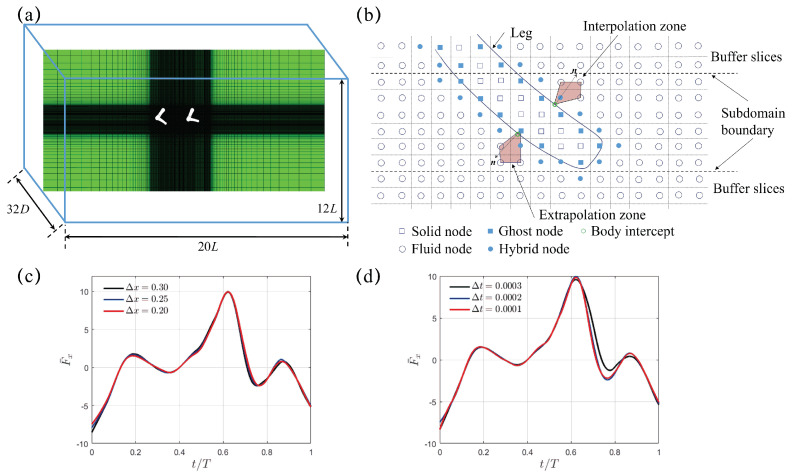
(**a**) Computational domain size and the stretched Cartesian mesh around the paddling legs. (**b**) Schematic of a subdomain with two buffer slices on each side in our parallel implementation. The shaded regions represent the stencil for local interpolation and extrapolation. (**c**) Mesh independency study with Δx=0.30 cm, 0.25 cm, and 0.20 cm. (**d**) Time independency study with Δt=0.0003 s, 0.0002 s, and 0.0001 s.

The legs are assumed to be rigid without deformation and the quadrupedal paddling system is tethered without displacement. Similar tethered models to ours are often used for the study of self-propulsion of fish swimming [35,36,37,38]. Assuming the structure is rigid, one-way coupling is employed to model the fluid-structure interaction, where the motion of the quadrupedal paddling significantly influences the fluid flow. However, structural deformation due to fluid forces is disregarded in this analysis. For spatial discretization, each leg is divided into 29,940 triangular surface elements with a total number of 14,974 nodes. The fluid is assumed to be Newtonian and incompressible. The governing equation of the flow is the unsteady three-dimensional viscous Navier-Stokes equation. Since the peak Reynolds number Re is on order of $O(10^{4})$, we do not consider turbulence effect in present work, although it may become necessary when the background environmental flow is chaotic or the robotic system size of quadrupedal paddling becomes larger. The fluid domain is represented by 20L×32D×12L cm^3^ rectangular bounding box and is divided by a 290×160×160 nonuniform Cartesian grid. Fine resolution with Δx=Δy=Δz=0.25 cm is used in the region around the four paddling legs (Figure A1a in Appendix A). No-slip and no-penetration boundary conditions are imposed on limb surface and zero velocity and zero normal pressure gradient boundary conditions are applied on the six outer faces of the fluid domain. Initially, the flowfield is static, and the flow is driven in motion by coordinated paddling. The density and dynamic viscosity of the fluid are, respectively, ρ=1 g/cm^3^ and μ=0.001 Pa·s. To ensure the numerical stability of the flow solver, the time step used in the simulation is Δt=0.0002 s, so that each paddling cycle of DR=50%, 33%, and 25% contains 4000, 6000 and 8000 time steps, respectively. The choice of mesh size Δx=0.25 cm and time step Δt=0.0002 s is based on the mesh and time step convergence studies described in Appendix A. The propulsion force discussed in this work is normalized by $\rho_{f}{U_{0}}^{2}W^{2}$, where a common limb tip velocity $U_{0}=0.42$ m/s under $\beta_{0}=40^{\circ }$ and DR=50%, and limb width *W* are chosen as our velocity and length reference, respectively. The viscous three-dimensional unsteady Navier-Stokes equation is numerically solved using a parallel in-house sharp-interface immersed-boundary method; more information on the computational implementation and mesh convergence study is provided in Appendix A.

## 3. Results

### 3.1. Hydrodynamics of a Single Paddling Leg

**Figure 2:**
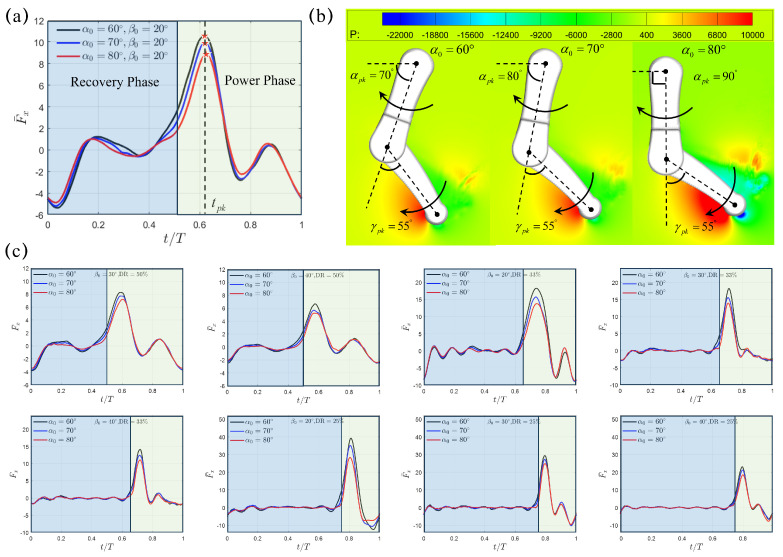
(**a**) The profiles of the transient thrust ${\bar{F}}_{x}\left(t\right)$ generated by a single paddling leg under $\alpha_{0}=60^{\circ }$, $70^{\circ }$, and $80^{\circ }$ at fixed $\beta_{0}=20^{\circ }$ and DR=50%. (**b**) The instantaneous pressure contour (Unit: 0.1 Pa) and the corresponding leg configuration with its upper- and lower-limb alignment at the peak thrust moment $t_{pk}$. The transient variation of angles (**c**) Transient thrust at different initial swing angle $\alpha_{0}$, swing amplitude $\beta_{0}$ and power phase ratio DR. Recovery and power phases are marked using blue and green windows, respectively.

We first conduct numerical simulations to investigate the hydrodynamics of a single paddling leg with the initial swing angle $\alpha_{0}\in \left[60^{\circ },70^{\circ },80^{\circ }\right]$ and swing amplitude $\beta_{0}\in \left[20^{\circ },30^{\circ },40^{\circ }\right]$ under three different power phase ratios DR=50%, 33% and 25%. The time duration of each paddling cycle is T=0.8 s, 1.2 s, and 1.6 s for $\beta_{0}=20^{\circ }$, $30^{\circ }$, and $40^{\circ }$, respectively. To avoid the influence caused by the initial condition that sets zero fluid velocity everywhere, simulation results from the 5th cycle is shown in Figure 2. Figure 2a presents the transient profile of the thrust ${\bar{F}}_{x}\left(t\right)$ with varying initial swing angle $\alpha_{0}\in [60^{\circ }$,$70^{\circ }$, $80^{\circ }]$ at a fixed swing amplitude of $\beta_{0}=20^{\circ }$. We can see that, although the paddling motion gives rise to thrust (${\bar{F}}_{x}\left(t\right)>0$) and drag (${\bar{F}}_{x}\left(t\right)<0$) in both recovery and power phases, most of the thrust is generated during the power phase. Due to the rapid backswing of the lower limb, ${\bar{F}}_{x}\left(t\right)$ first rises quickly from t=3.5 s (t=0.375T) before reaching a peak value ${\bar{F}}_{x,pk}$ at t=3.7 s (t=0.625T), it then experiences a fast drop as the backswing motion continues. As the initial swing angle $\alpha_{0}$ increases from $60^{\circ }$ to $70^{\circ }$ then to $80^{\circ }$, the corresponding peak thrust ${\bar{F}}_{x,pk}$ reduces from 10.53 to 9.90 then to 8.79. However, ${\bar{F}}_{x,pk}$ occurs almost at the same instant of $t_{pk}=3.7$ s, which corresponds to $t_{pk}=0.625T$ within a paddling cycle with period T=0.8 s for $\beta_{0}=20^{\circ }$. Within the complete paddling cycle, the average thrust ${\bar{F}}_{x,avg}$ also decreases from 0.75 to 0.59 then to 0.43 with increasing $\alpha_{0}=60^{\circ }$, $70^{\circ }$, and $80^{\circ }$. When the single leg is accelerated posteriorly, a high pressure region is generated on the back side of the lower limb, while leaving a negative-pressure region simultaneously in its front side (Figure 2b). This pressure difference across the lower limb for $\alpha_{0}=60^{\circ }$, $70^{\circ }$, and $80^{\circ }$ shares a similar pattern and leads to the so-called drag-based thrust. At the peak thrust moment $t_{pk}$, the orientation of the upper limb is different with $\alpha_{pk}=70^{\circ }$, $80^{\circ }$, and $90^{\circ }$ for $\alpha_{0}=60^{\circ }$, $70^{\circ }$ and $80^{\circ }$, respectively; however, the relative position of the lower limb with respect to the upper limb remains the same with $\gamma_{pk}=55^{\circ }$ (Figure 2b). Similar varying trends for ${\bar{F}}_{x,pk}$, $t_{pk}$, $\alpha_{pk}$, $\gamma_{pk}$ and ${\bar{F}}_{x,avg}$, as well as the pressure contour around the paddling leg at ${\bar{F}}_{x,pk}$, are also observed for the cases with larger swing angles $\beta_{0}=30^{\circ }$ and $40^{\circ }$. It can be seen that the average thrust ${\bar{F}}_{x,avg}$ drops with respect to both increasing initial swing angle $\alpha_{0}$ and swing amplitude $\beta_{0}$. The lowest ${\bar{F}}_{x,avg}$ is generated at the largest $\alpha_{0}=80^{\circ }$ and $\beta_{0}=40^{\circ }$. In Figure 2c, we also presented the profiles of transient thrust generated under other combinations of initial swing angle $\alpha_{0}$, swing amplitude $\beta_{0}$ and power phase ratio DR, which will be used for the discussion of inter-limb interaction later. In addition, we computed the thrust coefficient via $C_{T}=2F_{x,avg}/\rho_{f}{\bar{U}}^{2}L^{2}W$, where $\bar{U}$ is the average lower limb tip velocity of individual cases. Upon fixing $\beta_{0}$ and DR, a consistently higher $C_{T}$ can be achieved at smaller $\alpha_{0}$. Relevant results are summarized in Table 2.

### 3.2. Quadrupedal Paddling Using the Trotting Gait

**Figure 3:**
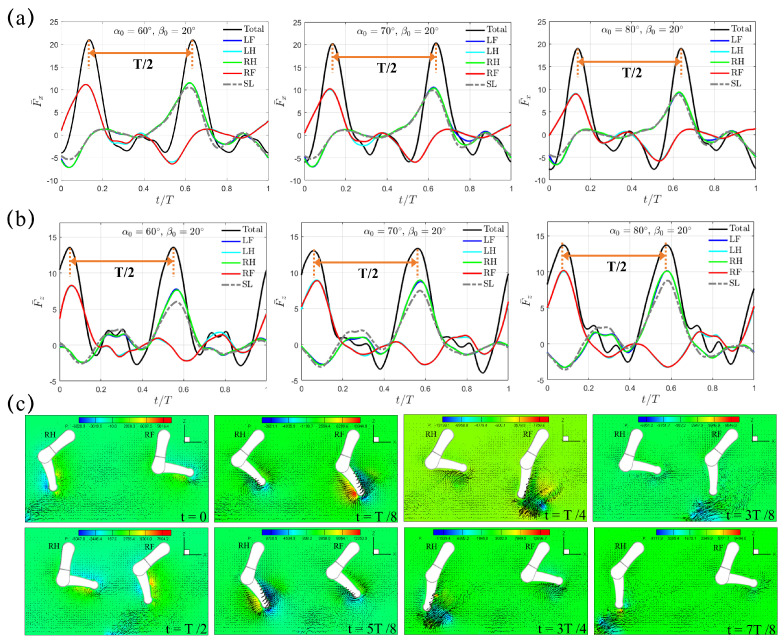
The transient profiles of (**a**) horizontal thrust ${\bar{F}}_{x}\left(t\right)$ and (**b**) vertical lift ${\bar{F}}_{z}\left(t\right)$ generated by the quadrupedal paddling using the trotting gait under $\beta_{0}=20^{\circ }$. Results from the single leg (SL) paddling are also included. (**c**) Velocity vectors along with pressure contour (Unit: 0.1 Pa) in the plane that cuts through RF and RH at t/T=0, 1/8, 1/4, 3/8, 1/2, 5/8, 3/4, and 7/8. Only every one out of two grid points is shown. An animation of the velocity field is also provided (Supplementary Materials available online).

To investigate the impact of quadrupedal paddling on total thrust, we next consider the trotting gait sequence, which is adopted by many terrestrial mammals. In this sequence, each pair of diagonal legs share the same movement, while the two legs on the same side have opposite movement. The duration of the power phase is equal to that of the recovery phase, which corresponds to a power phase ratio of DR=50%. Figure 3a,b show the transient profiles of the horizontal and vertical forces, ${\bar{F}}_{x}\left(t\right)$ and ${\bar{F}}_{z}\left(t\right)$, within a complete paddling cycle of T=0.8 s under $\beta_{0}=20^{\circ }$. Distinct from the single paddling leg situation, under the fixed swing amplitude of $\beta_{0}=20^{\circ }$, both ${\bar{F}}_{x}\left(t\right)$ and ${\bar{F}}_{z}\left(t\right)$ now demonstrate two peak values with an equal time interval of T/2. ${\bar{F}}_{x,pk}$ occurs at t=3.3 s (t=0.125T) and 3.7 s (t=0.625T), while ${\bar{F}}_{z,pk}$ is achieved at t=3.25 s (t=0.0625T) and 3.65 s (t=0.5625T), which corresponds to a slight 6.25% phase lead over ${\bar{F}}_{x,pk}$. When one pair of diagonal legs generates thrust in the power phase, the remaining two diagonal legs are in the recovery phase and have a counterproductive effect by generating drag (Figure 3a). Figure 3c shows the pressure and velocity fields in the plane that cuts through RF and RH at t/T=0, 1/8, 1/4, 3/8, 1/2, 5/8, 3/4, and 7/8. Using the length scale of W=4.65 cm and velocity scale of $MDPI\bar{U}=0.64$ m/s, the corresponding Reynolds number for this case is around $Re=3.0\times 10^{4}$. Vortex shedding from the tip of the lower limb as a result of its rapid paddling motion is conspicuous. During the first half of the trotting cycle t/T∈[0,1/2], RF is in the power phase and a counterclockwise vortex is produced near the tip of the lower limb when t/T=1/8 and 1/4. Meanwhile, RH is in the recovery phase and a clockwise vortex can be observed near its lower limb tip. During the latter half of the trotting cycle t/T∈[1/2,1], the vortex behavior that RF and RH have previously experienced is flipped, and clockwise and counterclockwise vortices are formed near the lower limb tips of RF and RH respectively.

As the initial swing angle $\alpha_{0}$ increases from $60^{\circ }$ to $70^{\circ }$ to $80^{\circ }$, the instants of ${\bar{F}}_{x,pk}$ and ${\bar{F}}_{z,pk}$, the time intervals between ${\bar{F}}_{x,pk}$ and ${\bar{F}}_{z,pk}$, and the time lead of ${\bar{F}}_{z,pk}$ over ${\bar{F}}_{x,pk}$ remain unchanged. However, the changes of ${\bar{F}}_{x,pk}$ and ${\bar{F}}_{z,pk}$ are in an opposite trend. ${\bar{F}}_{x,pk}$ reduces from 20.95 to 20.30 to 18.95 when $\alpha_{0}$ increases from $60^{\circ }$ to $70^{\circ }$ to $80^{\circ }$, respectively, while at the same time ${\bar{F}}_{z,pk}$ experiences a slight raise from 12.75 to 13.02 to 13.55 (Figure 3a,b). Similarly, ${\bar{F}}_{x,avg}$ drops from 3.34 to 2.76 to 1.83, while ${\bar{F}}_{z,avg}$ increases from 3.23 to 3.42 to 3.97. Within the power phase, two diagonal legs contribute equally to ${\bar{F}}_{x}\left(t\right)$. If the phase difference of T/2 in ${\bar{F}}_{x}\left(t\right)$ between the two pairs of diagonal legs is ignored, the magnitudes of horizontal and vertical forces of the four individual legs are almost identical, indicating that the four legs also contribute equally to the total forces of ${\bar{F}}_{x}\left(t\right)$ and ${\bar{F}}_{z}\left(t\right)$. Hereafter, we denote this mechanism of propulsion generation as the “Trotting Mode”.

**Figure 4:**
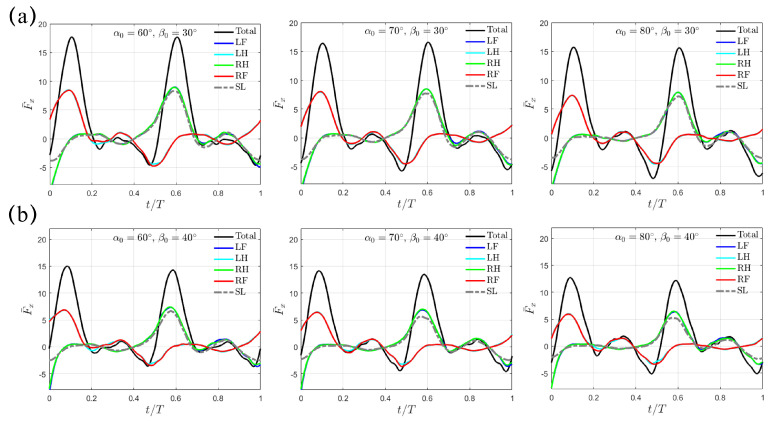
The transient profiles of thrust ${\bar{F}}_{x}\left(t\right)$ for swing amplitude (**a**) $\beta_{0}=30^{\circ }$ and (**b**) $\beta_{0}=40^{\circ }$. Results from the SL paddling are also included.

As shown in Figure 4, similar trends for the “Trotting Mode” mentioned above still remain for the cases with larger swing amplitudes of $\beta_{0}=30^{\circ }$ and $40^{\circ }$. For $\beta_{0}=30^{\circ }$, the average thrust ${\bar{F}}_{x,avg}$ reduces from 2.80 to 2.39 to 1.90 for $\alpha_{0}=60^{\circ }$, $70^{\circ }$ and $80^{\circ }$; for $\beta_{0}=40^{\circ }$, it also drops from 2.54 to 2.24 to 1.90. Overall, the combination with smaller magnitudes of $\alpha_{0}=60^{\circ }$ and $\beta_{0}=20^{\circ }$ provides the highest average propulsion thrust in the “Trotting Mode”.

With the data available for a single paddling leg as listed in Table 2, we are able to quantitatively evaluate the inter-limb interaction and thus trotting gait effectiveness under different combinations of initial swing angle and swing amplitude $\left(\alpha_{0},\beta_{0}\right)$. To do so, we compute the force ratio of the average thrust ${\bar{F}}_{x}/4{\bar{F}}_{SL}$ between the quadrupedal and single-leg paddling motions. In Table 3, we can see that the inter-limb interaction in the trotting gait consistently enhances the thrust with ${\bar{F}}_{x}/4{\bar{F}}_{SL}>1$, which indicates that the hydrodynamic benefit of enhanced thrust is successfully captured through the cooperation of diagonal legs. For example, ${\bar{F}}_{x}/4{\bar{F}}_{SL}$ can reach 1.26 with $\left(\alpha_{0},\beta_{0}\right)=\left(70^{\circ },40^{\circ }\right)$, which means that the average thrust ${\bar{F}}_{x}$ of an individual leg in this particular trotting gait is 1.26 times the magnitude of that generated by the single paddling leg with the same combination of $\left(\alpha_{0},\beta_{0}\right)$. From the comparison in Figure 3a between the single-leg and quadrupedal paddling, it can be seen that coordinated paddling slightly increases thrust but significantly reduces drag. From the comparison in Figure 3b, the 4-leg paddling significantly reduce the magnitude of vertical force.

### 3.3. Quadrupedal Paddling Using the Diagonal Sequence Gait

**Figure 5:**
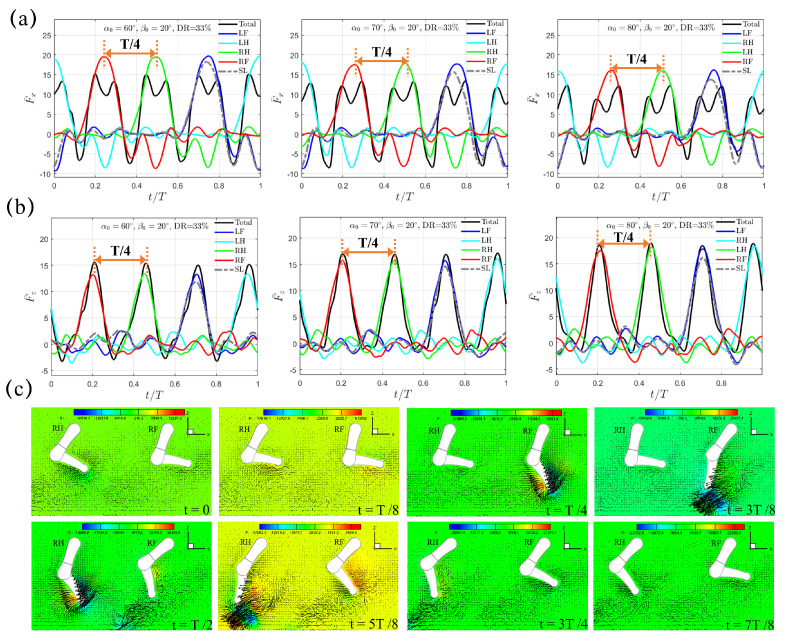
The transient profiles of (**a**) thrust ${\bar{F}}_{x}\left(t\right)$ and (**b**) vertical force ${\bar{F}}_{z}\left(t\right)$ generated by the quadrupedal paddling using the diagonal sequence gait when $\beta_{0}=20^{\circ }$ and DR=33%. Results from the SL paddling are also included. (**c**) Velocity vectors along with pressure contour (Unit: 0.1 Pa) in the plane that cuts through RF and RH at t/T=0, 1/8, 1/4, 3/8, 1/2, 5/8, 3/4, and 7/8. Only every one out of two grid points is shown. An animation of the velocity field is also provided (Supplementary Materials available online).

Next, we consider the diagonal sequence gait that is actually used when swimming by dogs of different breeds [34]. In this sequence (LF→LH→RF→RH), The power stroke of the hindlimb on one side of the body is succeeded by that of the opposite side’s forelimb. Unlike the trotting gait with DR=50%, two shorter power phases with DR=33% and 25% are considered with the intuition that faster backswing motion should be beneficial for larger thrust generation. In reality, DR=33% is representative for dogs of different breeds during swimming. For DR=33%, the average lower limb tip velocity $\bar{U}$ in the power phase is 0.96, 0.73, and 0.60 m/s for $\beta_{0}=20^{\circ }$, $30^{\circ }$, and $40^{\circ }$, respectively; for DR=25%, it becomes 1.34, 1.03, and 0.84 m/s for $\beta_{0}=20^{\circ }$, $30^{\circ }$, and $40^{\circ }$, respectively. Figure 5a,b present the transient profiles of horizontal and vertical forces, ${\bar{F}}_{x}\left(t\right)$ and ${\bar{F}}_{z}\left(t\right)$, generated when $\beta_{0}=20^{\circ }$ and DR=33%. It can be seen that, both ${\bar{F}}_{x}\left(t\right)$ and ${\bar{F}}_{z}\left(t\right)$ exhibit a compact four-peak waveform that is equally distributed within the gait cycle with interval T/4, which is in contrast to the two-peak waveform observed in the “Trotting Mode”. In diagonal sequence gait, the power phase of each leg contributes to one wave of thrust ${\bar{F}}_{x}\left(t\right)$. Moreover, the thrust ${\bar{F}}_{x}\left(t\right)$ generated by one individual leg is significantly impaired by the induced drag from the preceding leg leaving the current power phase and entering the following recovery phase, thus leaving an M-shaped profile around ${\bar{F}}_{x,pk}$. This represents a different hindering mechanism (denoted as the “Hindering Mode” hereafter) of ${\bar{F}}_{x}\left(t\right)$ in Figure 5a as compared with that generated in the “Trotting Mode” in Figure 3a. Under the same initial swing angle $\alpha_{0}$ and swing amplitude $\beta_{0}$, even though the magnitude of peak thrust ${\bar{F}}_{x,pk}$ in the “Hindering Mode” is lower than that in the “Trotting Mode”, the average thrust ${\bar{F}}_{x,avg}$ in the “Hindering Mode” is much higher due to its shorter power phase and faster leg motion. For example, when $\alpha_{0}=60^{\circ }$ and $\beta_{0}=20^{\circ }$, ${\bar{F}}_{x,pk}$ is 20.95 and 14.77 for the “Trotting Mode” and the “Hindering Mode”, respectively, while the corresponding ${\bar{F}}_{x,avg}$ is 3.34 and 6.12, respectively, which represents a large increment of about 83.23%. A slight phase lead of t=0.05 s (*t* = 0.0625*T*), which is identical to that of the trotting gait, is observed between ${\bar{F}}_{x}\left(t\right)$ and ${\bar{F}}_{z}\left(t\right)$. Due to its accelerated power phase, the average lower limb tip velocity in power phase increases to $\bar{U}=0.96$ m/s, yielding a Reynolds number $Re=4.5\times 10^{4}$. Within one complete cycle of diagonal sequence gait, counterclockwise vortex is shed from the lower limb tip by following the LF→LH→RF→RH sequence during power phase. For example, a counterclockwise vortex near the lower limb tip of RF and RH is observed at t/T=1/4 and 3/8, and t/T=1/2 and 5/8, respectively (Figure 5c).

The varying trends for the instants of ${\bar{F}}_{x,pk}$ and ${\bar{F}}_{z,pk}$, the time intervals between ${\bar{F}}_{x,pk}$ and ${\bar{F}}_{z,pk}$, and the slight time lead of ${\bar{F}}_{z,pk}$ over ${\bar{F}}_{x,pk}$ are maintained when we increase the initial swing angle $\alpha_{0}$ to $70^{\circ }$ and then $80^{\circ }$. ${\bar{F}}_{x,pk}$ reduces from 14.77 to 13.29 to 12.22 and ${\bar{F}}_{x,avg}$ also reduces from 6.12 to 5.12 to 4.41; while ${\bar{F}}_{z,pk}$ increases from 14.93 to 16.95 to 18.65 with increasing ${\bar{F}}_{z,avg}$ from 4.91 to 5.54 to 6.24. At the moment of peak thrust ${\bar{F}}_{x,pk}$, the corresponding $\alpha_{pk}$ is $70^{\circ }$, $80^{\circ }$, and $90^{\circ }$ for $\alpha_{0}=60^{\circ }$, $70^{\circ }$, and $80^{\circ }$, respectively, which are identical with that from the trotting gait.

**Figure 6:**
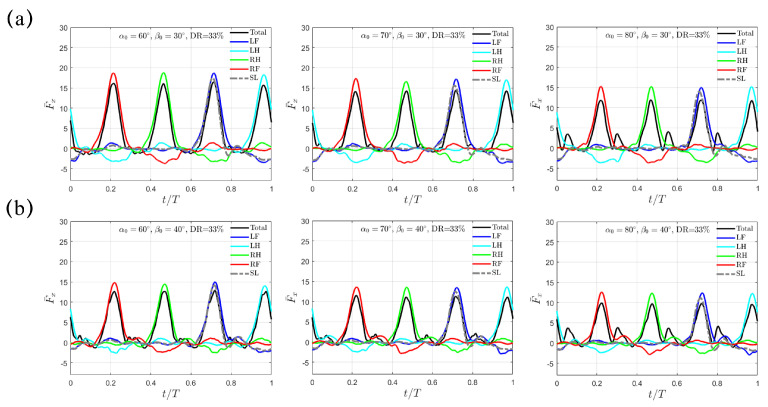
The transient profiles of thrust ${\bar{F}}_{x}\left(t\right)$ for swing amplitude (**a**) $\beta_{0}=30^{\circ }$ and (**b**) $\beta_{0}=40^{\circ }$ when DR=33%. Results from the SL paddling are also included.

In Figure 6, the transient profiles of ${\bar{F}}_{x}\left(t\right)$ are presented for the cases with larger swing amplitudes of $\beta_{0}=30^{\circ }$ and $40^{\circ }$. The varying trend is similar to the situation with $\beta_{0}=20^{\circ }$ discussed above. For $\beta_{0}=30^{\circ }$, ${\bar{F}}_{x,avg}$ decreases from 4.37 to 3.81 to 3.23 for $\alpha_{0}=60^{\circ }$, $70^{\circ }$ and $80^{\circ }$; for $\beta_{0}=40^{\circ }$, it further drops from 3.41 to 3.20 to 2.89, respectively. At the same $\alpha_{0}$ and $\beta_{0}$, ${\bar{F}}_{x,avg}$ is evidently higher than that generated with the trotting gait. In addition, different from the M-shaped profile left around ${\bar{F}}_{x,pk}$ under $\beta_{0}=20^{\circ }$, the thrust impairment is gentler as $\beta_{0}$ increases to $30^{\circ }$ and $40^{\circ }$. Thus, a peak value in total thrust ${\bar{F}}_{x,pk}$ is still observable. Like the trotting gait, $\alpha_{pk}$ increases with increasing $\alpha_{0}$ and $\beta_{0}$, while $\gamma_{pk}$ maintains the same with respect to $\alpha_{0}$ but only decreases as $\beta_{0}$ increases. However, for the same swing amplitude $\beta_{0}$, the values of $\alpha_{pk}$ and $\gamma_{pk}$ remain identical to those in the trotting gait.

**Figure 7:**
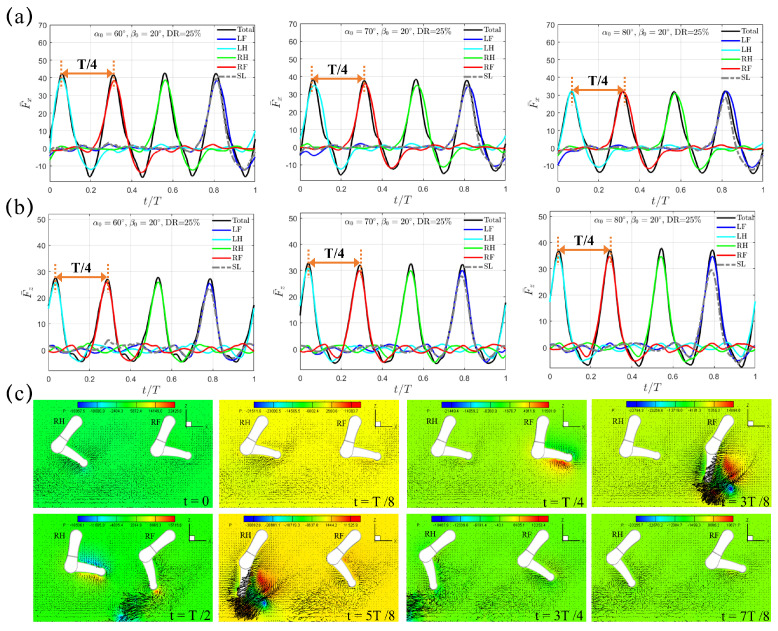
The transient profiles of (**a**) thrust ${\bar{F}}_{x}\left(t\right)$ and (**b**) vertical force ${\bar{F}}_{z}\left(t\right)$ generated by the quadrupedal paddling using the diagonal sequence gait when $\beta_{0}=20^{\circ }$ and DR=25%. Results from the SL paddling are also included. (**c**) Velocity vectors along with pressure contour (Unit: 0.1 Pa) in the plane that cuts through RF and RH at t/T=0, 1/8, 1/4, 3/8, 1/2, 5/8, 3/4, and 7/8. Only every one out of two grid points is shown. An animation of the velocity field is also provided (Supplementary Materials available online).

Next, we consider an even shorter power phase using the diagonal sequence gait with DR=25%. The average lower limb tip velocity $\bar{U}$ in the power phase is about 1.34 m/s, which yields a Reynolds number of $Re=6.2\times 10^{4}$. Compared to that of DR=33%, ${\bar{F}}_{x}\left(t\right)$ and ${\bar{F}}_{z}\left(t\right)$ increase significantly, as shown in Figure 7a,b. As $\alpha_{0}$ increases from $60^{\circ }$, $70^{\circ }$, to $80^{\circ }$, ${\bar{F}}_{x,avg}$ increases to 9.36, 8.35 and 6.42 as compared to 6.12, 5.12, and 4.41 when DR=33%. Although ${\bar{F}}_{x}\left(t\right)$ and ${\bar{F}}_{z}\left(t\right)$ still exhibit a compact four-peak waveform that is equally spaced within one paddling cycle with a uniform interval of T/4, the drag caused by the preceding leg leaving the current power phase and entering the following recovery phase in the “Hindering Mode” disappears. Instead, the four legs now contribute independently in LH→RF→RH→LF sequence to the total thrust ${\bar{F}}_{x}\left(t\right)$. For instance, the paddling of LH generates thrust (t/T∈[0,1/8]) and a drag (t/T∈[1/8,1/4]) during the first quarter of the whole diagonal sequence gait; outside of the time interval t/T∈[0,1/4], the contribution of LH to ${\bar{F}}_{x}\left(t\right)$ becomes negligible. Thus, we denote this mechanism of thrust generation as the “Separate Mode”. Compared to the case of DR=33% with $\alpha_{0}=60^{\circ }$, both $\alpha_{pk}$ and $\gamma_{pk}$ share the same value, resulting in a configuration where the upper-lower limb alignment is tilted forward. Thus, the reduced power phase ratio from DR=33% to 25% does not change the arrangement between the upper and lower limbs at $t_{pk}$. We again present the velocity field in the slice that cuts through RF and RH in Figure 7c. Like the previous situation with DR=33%, a counterclockwise vortex is shed from the lower limb tip in LF→LH→RF→RH sequence during power phase. At t/T=1/4 and 3/8, and t/T=1/2 and 5/8, such counter-clockwise vortex is clearly observed near the lower limb tips of RF and RH (Figure 7c).

**Figure 8:**
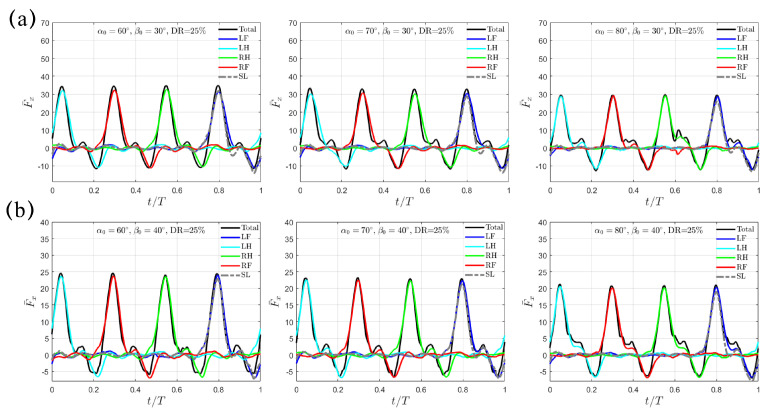
The transient profiles of thrust ${\bar{F}}_{x}\left(t\right)$ for swing amplitude (**a**) $\beta_{0}=30^{\circ }$ and (**b**) $\beta_{0}=40^{\circ }$ when DR=25%. Results from the SL paddling are also included.

As the swing amplitude $\beta_{0}$ increases to $30^{\circ }$ and $40^{\circ }$, the “Separate Mode” discussed above for $\beta_{0}=20^{\circ }$ still holds (Figure 8). Like the “Trotting Mode” and the “Hindering Mode”, the thrust ${\bar{F}}_{x}\left(t\right)$ decreases as the initial swing angle $\alpha_{0}$ increases. For $\beta_{0}=30^{\circ }$, ${\bar{F}}_{x,avg}$ is 6.62, 6.28, and 5.23 for $\alpha_{0}=60^{\circ }$, $70^{\circ }$, and $80^{\circ }$, respectively; for $\beta_{0}=40^{\circ }$, it decreases further to 5.03, 4.81, and 4.49 respectively. Again, $\alpha_{pk}$ increases with both increasing $\alpha_{0}$ and $\beta_{0}$, while $\gamma_{pk}$ remains unchanged with respect to $\alpha_{0}$ but only decreases as $\beta_{0}$ increases. Taking all the cases with DR=33% and 25% together, we note that a leg configuration with a larger forward-tilted angle and a flatter upper-lower limb alignment at $t_{pk}$ indicates a lower thrust generation.

In Table 3, we also present the force ratio of ${\bar{F}}_{x}/4{\bar{F}}_{SL}$ pertaining to the diagonal sequence gait. In general, inter-limb interaction again enhances the thrust with ${\bar{F}}_{x}/4{\bar{F}}_{SL}>1$, with the exception of $\left(\alpha_{0},\beta_{0},DR\right)=\left(60^{\circ },20^{\circ },25\%\right)$. When the power phase ratio is reduced to DR=33% and 25%, even higher value of ${\bar{F}}_{x}/4{\bar{F}}_{SL}$ can be achieved. For example, ${\bar{F}}_{x}/4{\bar{F}}_{SL}$ can achieve 1.87 under $\left(\alpha_{0},\beta_{0},DR\right)=\left(80^{\circ },20^{\circ },25\%\right)$, which means that the average thrust ${\bar{F}}_{x}$ of an individual leg from this particular diagonal sequence gait is 1.87 times the magnitude of that generated by a single leg under the same combination of $\left(\alpha_{0},\beta_{0},DR\right)$. Hence, the hydrodynamics of quadrupedal paddling is not necessarily a simple linear superposition with phase shift of those from single-leg paddling, and the effect of inter-limb interaction needs to be put into consideration during the design of paddling gaits. Since only three different values of $\alpha_{0}$, $\beta_{0}$ and DR are considered in the present study, which represents the limitation of the present work, a closer investigation of the detailed correlation between the force ratio ${\bar{F}}_{x}/4{\bar{F}}_{SL}$ and the kinematic parameters is not straightforward with limited data and thus is deferred to a future study that can also incorporate geometry optimization and a comparison of energy expenditure with other propulsion mechanisms.

### 3.4. Quadrupedal Paddling Using the Lateral Sequence Gait

Using the same power phase ratio of DR=25% and 33%, we continue our study to consider the lateral sequence gait and investigate how the hydrodynamics is affected by the paddling sequence. The lateral gait has the hindleg contact of the ground followed by the contact of the foreleg on the same side, and thus is directly opposite to the diagonal sequence gait. Our simulation results reveal that the transient thrust generated by the lateral sequence gait is almost the same as that generated by the diagonal sequence gait except that the paddling movement now follows LF→RH→RF→LH sequence. Cases with DR=25% experience the “Separate Mode”, in which each leg’s contribution to the total thrust ${\bar{F}}_{x}\left(t\right)$ is limited to its own paddling phase with a time duration of DR·T. On the other hand, cases with DR=33% experience the “Hindering Mode”, in which the thrust ${\bar{F}}_{x}\left(t\right)$ contributed by an individual leg is significantly reduced by the induced drag of the preceding leg leaving its current power phase and entering the following recovery phase. The thrust generation is closely related to the pressure difference across the lower limb during the backswing in the power phase. In general, the “Hindering Mode” and the “Separate Mode” produce higher thrust efficiency than the “Trotting Mode” through inter-limb interaction. However, thrust efficiency may experience degradation under certain combinations of coefficients, such as $\left(\alpha_{0},\beta_{0},DR\right)=\left(60^{\circ },20^{\circ },25\%\right)$, as presented in Table 3.

### 3.5. Moments and Energetics of Quadrupedal Paddling

**Figure 9:**
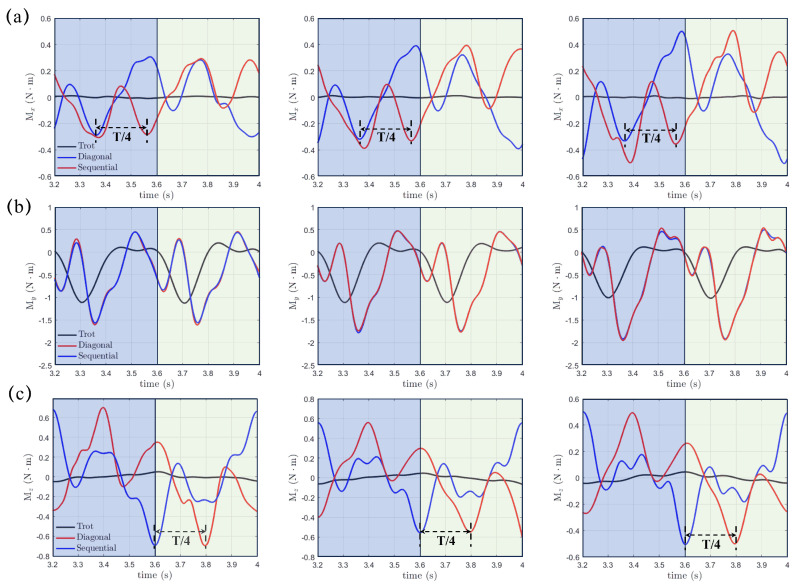
The transient profiles of hydrodynamic moments of (**a**) $M_{x}\left(t\right)$, (**b**) $M_{y}\left(t\right)$ and (**c**) $M_{z}\left(t\right)$ generated in both diagonal and lateral sequence gaits when DR=33% and comparison with that from the trotting gait.

**Figure 10:**
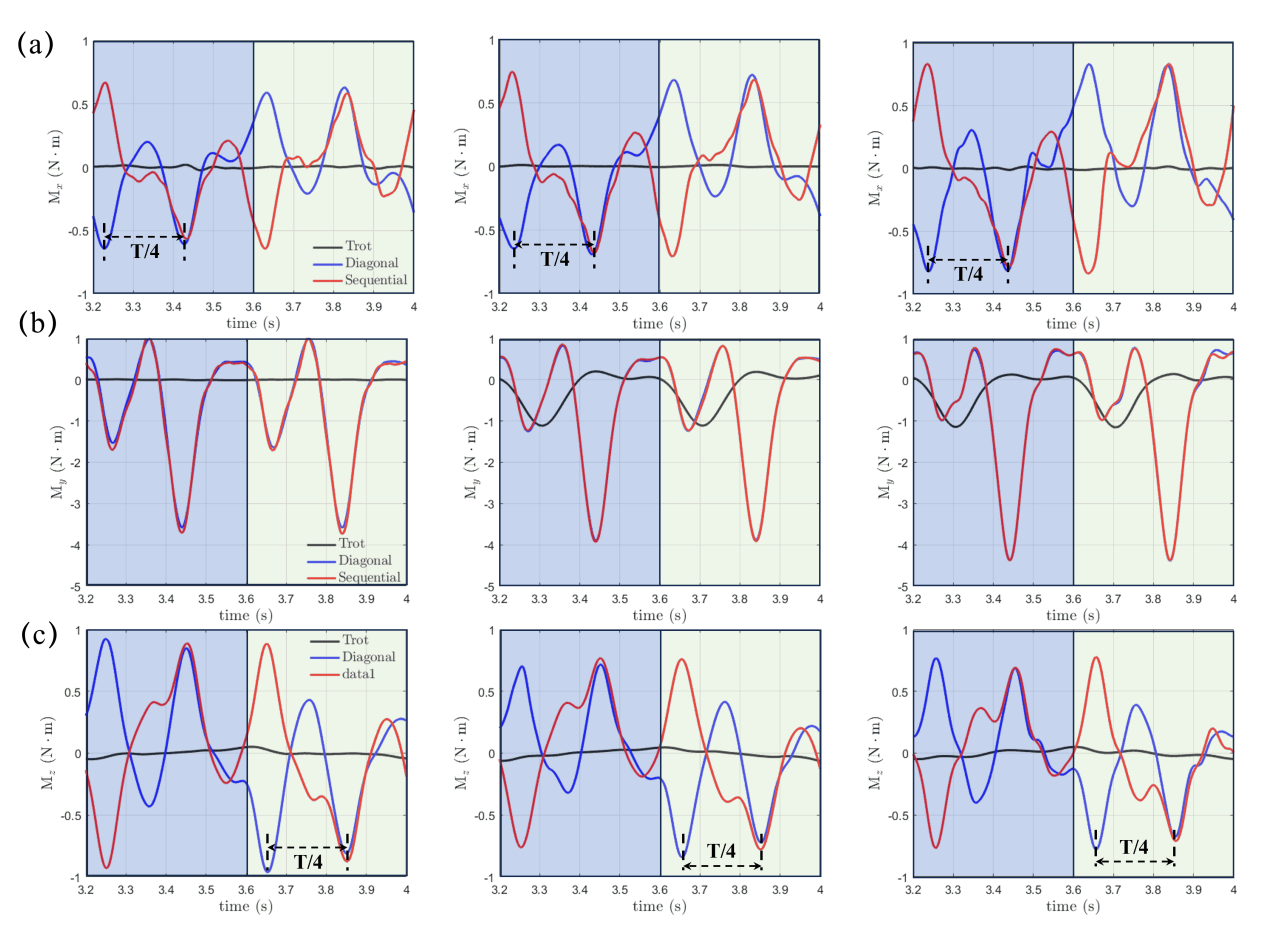
The transient profiles of moments of (**a**) $M_{x}\left(t\right)$, (**b**) $M_{y}\left(t\right)$ and (**c**) $M_{z}\left(t\right)$ generated in both diagonal and lateral sequence gaits when DR=25% and comparison with that from the trotting gait.

After obtaining detailed information about the computed hydrodynamic forces, we continue our study by examining the hydrodynamic moments, which can be expressed by

$$M_{x}=\int_{\Gamma }\left(F_{z}\cdot y-F_{y}\cdot z\right)d\Gamma ,\;\;M_{y}=\int_{\Gamma }\left(F_{x}\cdot z-F_{z}\cdot x\right)d\Gamma ,\;\;M_{z}=\int_{\Gamma }\left(F_{y}\cdot x-F_{x}\cdot y\right)d\Gamma$$

where $M_{x}$, $M_{y}$, and $M_{z}$ might instigate rolling, pitching, and yawing motions and significantly impact the stability of the real robotic dog paddling system. The origin O, as shown in Figure 1, is selected as the center, and the integration for moments is performed on the fluid-structure interface Γ. Figure 9 demonstrates the moments $M_{x}$, $M_{y}$, and $M_{z}$ with varying initial swing angle ($\alpha_{0}=60^{\circ }$, $70^{\circ }$, and $80^{\circ }$) under a fixed swing amplitude $\beta_{0}=20^{\circ }$ and power phase ratio DR=33%. The results of the trotting gait with DR=50% are also included for comparison. We can see that $M_{x}$, $M_{y}$, and $M_{z}$ in the diagonal and lateral sequence gaits are very close to each other, which indicates a quite similar stability behavior, even though a phase difference of t=T/4 does exist for both $M_{x}$ and $M_{z}$. In contrast, $M_{x}$, $M_{y}$, and $M_{z}$ generated in the trotting gait are much smaller, especially for $M_{x}$ and $M_{z}$. Due to the symmetry of the paddling motion with respect to the y=0 plane for diagonal and lateral sequence gaits within a complete gait cycle, the profiles of $M_{x}$ and $M_{z}$ in the power and recovery phases are also symmetric about y=0 with average values $M_{x,avg}$ and $M_{z,avg}$ equal to zero, as shown in Figure 9a,c. Thus, the four-leg paddling system would experience sustainable roll and yaw vibrations but is overall stable. In contrast, an overall negative $M_{y,avg}$ is detected throughout the entire paddling cycle in all three distinct gaits. As the initial swing angle $\alpha_{0}$ increases, the magnitudes of both $M_{x}$ and $M_{y}$ increase while that of $M_{z}$ reduces. Similar tendencies for $M_{x}$, $M_{y}$ and $M_{z}$ persist at larger swing amplitudes of $\beta_{0}=30^{\circ }$ and $40^{\circ }$. In Figure 10, we also present the $M_{x}$, $M_{y}$, and $M_{z}$ profiles for cases with even shorter power phase under DR=25%. Clearly, compared with the case of DR=33%, the magnitudes of $M_{x}$, $M_{y}$, and $M_{z}$ become consistently larger for a fixed combination of $\alpha_{0}$ and $\beta_{0}$. Overall, the “Hindering Mode” (DR=33%) results in larger pitching, rolling, and yawing moments than the “Trotting Mode”. Moreover, in the “Separate Mode”, the hydrodynamic moments are even higher when DR=25%. Therefore, achieving larger thrust generation is not without expense. The associated stronger vibrations in hydrodynamic moments will enhance the control difficulty of the robotic quadrupedal paddling system. In real robotic systems of quadrupedal paddling, we can use appropriate sensors to receive the real-time three-dimensional vibration signals and meanwhile feedback control algorithms can be developed to attenuate strong vibrations to a desired level.

**Figure 11:**
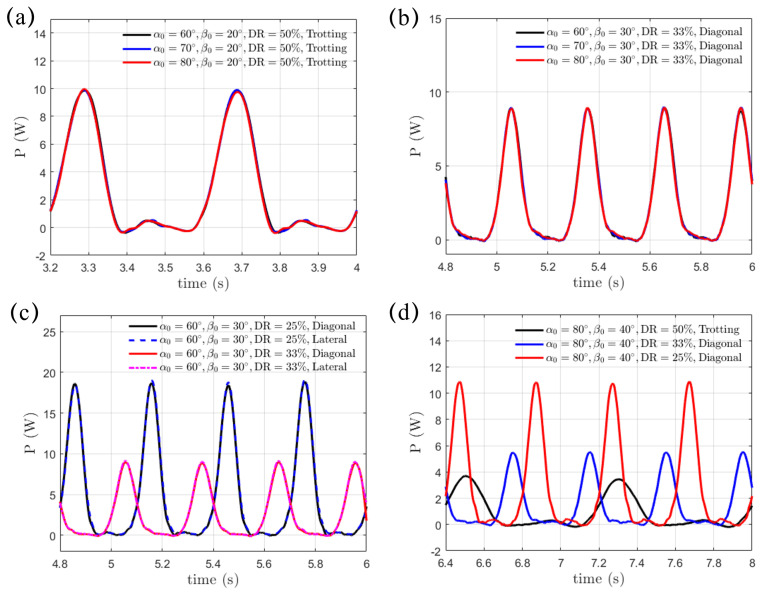
Identical power consumption in the (**a**) trotting gait and (**b**) diagonal sequence gait with varying initial swing angle $\alpha_{0}\in \left[60^{\circ },70^{\circ },80^{\circ }\right]$. (**c**) The diagonal and lateral sequence gaits share the same power consumption under fixed swing amplitude $\beta_{0}$ and power phase ratio DR. (**d**) The power expenditure increases with the reducing DR from 50% to 33% and 25%.

Finally, we present the transient profiles of power, which can be expressed by

$$P\left(t\right)=-\int_{\Gamma }F(t)\cdot u(t)d\Gamma$$

where P(t) is exerted on the fluid by the paddling legs, as shown in Figure 11. The minus sign is added here since F(t) represents the hydrodynamic force vector exerted on the paddling legs by the surrounding fluid, while u(t) is its moving velocity vector. Upon a given paddling gait, swing amplitude $\beta_{0}$ and power phase ratio DR, the transient power P(t) is independent of the initial swing angle $\alpha_{0}$, as shown in Figure 11a,b. Furthermore, the diagonal and lateral sequence gaits exhibit the same transient power profile. This indicates that, under the same swing amplitude $\beta_{0}$ and power phase ratio DR, the gait sequence, be it diagonal or lateral, has no impact on the power consumption (Figure 11c). Additional power is required when the power phase is shortened in the “Hindering Mode” (DR=33%) and the “Separate Mode” (DR=25%) for enhanced propulsion, as shown in Figure 11d. As DR decreases from 50% to 33% and 25% but with $\alpha_{0}=80^{\circ }$ and $\beta_{0}=40^{\circ }$ fixed, we integrate the transient power P(t) over a complete paddling cycle, and the resultant requested work are 1.37 J, 2.40 J, and 4.37 J, respectively. As a result, increased energy consumption becomes essential to achieve more effective propulsion. We also present the dimensionless power coefficient ${\bar{P}}_{avg}$, which is normalized by $\rho_{f}{\bar{U}}^{3}L_{2}W/2$, in Table 4. Again, for a given DR and $\beta_{0}$, ${\bar{P}}_{avg}$ remains unaffected by $\alpha_{0}$. Under fixed $\beta_{0}$, ${\bar{P}}_{avg}$ drops discernibly with increasing DR. However, ${\bar{P}}_{avg}$ with $\beta_{0}=30^{\circ }$ and $40^{\circ }$ under the same DR are very close to each other. The excessive power expenditure at smaller DR may impact the long-term performance of quadruped robots, influencing endurance, efficiency, and operational reliability. An appropriate gait strategy is therefore critical to help optimize energy use and extend mission duration. In addition, a lightweight yet durable structural design and a streamlined body shape also help minimize drag, enhance locomotion efficiency and reduce energy loss.

## 4. Discussion

In this work, we numerically investigate the effect of gait sequence, initial swing angle, swing amplitude, and power phase ratio on thrust generation of a bio-inspired robotic dog paddling model. For the trotting gait, the thrust engendered by one set of diagonal legs in the power phase is compromised by the other set of diagonal legs. Enhanced thrust is achieved through the reduced power phase in diagonal and lateral sequence gaits. The “Hindering Mode” is observed when the thrust from one paddling leg in the power phase is undermined by the preceding leg leaving its current power phase and entering the following recovery phase; while in the “Separate Mode”, the thrust contribution from each of the four individual legs is uniformly distributed within one paddling cycle and is almost independent to each other. In almost all cases, the interaction between limbs positively impacts thrust generation and generates a total thrust that is more than quadruple the thrust of a single leg. When implementing diagonal or lateral sequence gaits for propulsion in real-world robotic systems, particularly for lowerDR, it is essential to develop adequate feedback control strategies based on real-time vibration signals from sensors. These strategies are capable of attenuating the larger oscillatory pitching, rolling, and yawing moments to guarantee stability. Meanwhile, additional energy expenditure, which is independent of both initial swing angle and paddling sequence (whether diagonal or lateral), becomes inevitable.

## Figures and Tables

**Table 1:** Previous relevant CFD research on leg paddling.

Authors	Year	Leg Number	Simulation Approach	Application Type
Zhang, Calvin, et al. [32]	2014	4	Immersed Boundary Method	NA
Chen, Gang, et al. [31]	2021	2	Sliding/dynamic mesh	Fluent
Baines, Robert, et al. [33]	2022	4	SIMPLEC solver	Fluent

**Table 2:** Values of $\bar{U}$, $\alpha_{pk}$, $\gamma_{pk}$, ${\bar{F}}_{x,pk}$, ${\bar{F}}_{x,avg}$ and $C_{T}$ at swing amplitude $\beta_{0}\in \left[20^{\circ },30^{\circ },40^{\circ }\right]$, initial swing angle $\alpha_{0}\in \left[60^{\circ },70^{\circ },80^{\circ }\right]$ and power phase ratio of power phase DR∈[25%,33%,50%].

$\left(\beta_{0},\alpha_{0},\mathrm{DR}\right)$	$\bar{U}$ (m/s)	*T* (s)	$t_{\mathrm{pk}}\;\left(T\right)$	$\left(\alpha_{\mathrm{pk}},\beta_{\mathrm{pk}}\right)$	${\bar{F}}_{x,\mathrm{pk}}$	${\bar{F}}_{x,\mathrm{avg}}$	$C_{T}$
(20,60,50)	0.64	0.8	0.625	(70,55)	10.53	0.75	0.24
(20,70,50)	0.64	0.8	0.625	(80,55)	9.90	0.59	0.19
(20,80,50)	0.64	0.8	0.625	(90,55)	8.79	0.43	0.14
(30,60,50)	0.49	1.2	0.583	(80,45)	8.26	0.62	0.35
(30,70,50)	0.49	1.2	0.583	(90,45)	7.71	0.57	0.32
(30,80,50)	0.49	1.2	0.583	(100,45)	7.15	0.47	0.26
(40,60,50)	0.42	1.6	0.581	(90,35)	6.62	0.53	0.41
(40,70,50)	0.42	1.6	0.581	(100,35)	5.71	0.45	0.34
(40,80,50)	0.42	1.6	0.581	(110,35)	5.37	0.40	0.30
(20,60,33)	0.96	0.8	0.740	(70,55)	18.24	1.27	0.19
(20,70,33)	0.96	0.8	0.740	(80,55)	15.68	0.89	0.13
(20,80,33)	0.96	0.8	0.740	(90,55)	13.75	0.72	0.11
(30,60,33)	0.73	1.2	0.710	(80,45)	17.50	0.82	0.21
(30,70,33)	0.73	1.2	0.710	(90,45)	15.57	0.70	0.18
(30,80,33)	0.73	1.2	0.710	(100,45)	13.95	0.54	0.14
(40,60,33)	0.60	1.6	0.715	(90,35)	14.06	0.73	0.27
(40,70,33)	0.60	1.6	0.715	(100,35)	12.38	0.56	0.21
(40,80,33)	0.60	1.6	0.715	(110,35)	11.11	0.42	0.16
(20,60,25)	1.34	0.8	0.810	(70,55)	39.32	2.35	0.18
(20,70,25)	1.34	0.8	0.810	(80,55)	34.98	1.80	0.14
(20,80,25)	1.34	0.8	0.810	(90,55)	28.54	0.86	0.06
(30,60,25)	1.03	1.2	0.800	(80,45)	31.81	1.31	0.17
(30,70,25)	1.03	1.2	0.800	(90,45)	28.68	1.17	0.15
(30,80,25)	1.03	1.2	0.800	(100,45)	25.86	0.84	0.11
(40,60,25)	0.84	1.6	0.800	(90,35)	22.80	1.22	0.23
(40,70,25)	0.84	1.6	0.800	(100,35)	21.08	1.11	0.21
(40,80,25)	0.84	1.6	0.800	(110,35)	18.95	0.76	0.15

**Table 3:** Values of ${\bar{F}}_{x}/4{\bar{F}}_{SL}$ under different combinations of initial swing angle and swing amplitude $\left(\alpha_{0},\beta_{0}\right)$. SL stands for single leg.

${\bar{F}}_{x}/4{\bar{F}}_{\mathrm{SL}}$	Trotting	Diagonal,DR=33%	Diagonal,DR=25%
(60,20)	1.12	1.20	1.00
(70,20)	1.18	1.42	1.16
(80,20)	1.09	1.52	1.87
(60,30)	1.13	1.33	1.26
(70,30)	1.06	1.37	1.34
(80,30)	1.02	1.50	1.56
(60,40)	1.20	1.17	1.04
(70,40)	1.26	1.44	1.20
(80,40)	1.19	1.71	1.47

**Table 4:** Values of power coefficient ${\bar{P}}_{avg}$ under different combinations of initial swing angle and swing amplitude $\left(\alpha_{0},\beta_{0}\right)$.

${\bar{P}}_{\mathrm{avg}}$	Trotting	Diagonal,DR=33%	Diagonal,DR=25%
(60,20)	3.50	1.72	1.25
(70,20)	3.50	1.72	1.25
(80,20)	3.50	1.72	1.25
(60,30)	4.33	2.41	1.60
(70,30)	4.33	2.41	1.60
(80,30)	4.33	2.41	1.60
(60,40)	4.28	2.38	1.62
(70,40)	4.28	2.38	1.62
(80,40)	4.28	2.38	1.62

## Data Availability

The original contributions presented in this study are included in the article/Supplementary Material. Further inquiries can be directed to the corresponding authors.

## Supplementary Materials

The following supporting information can be downloaded at: https://www.mdpi.com/article/10.3390/biomimetics10030148/s1.

## Appendix A

The numerical approach and verification details of the sharp-interface immersed-boundary method based on the Cartesian grid for the flow utilized in this work have been expounded previously [39,40]. Successful applications to biological systems involve the fluid-structure interaction of heart valves [41,42] and vocal fold vibrations [43]. Furthermore, a parallel algorithm based on domain decomposition has been implemented to accelerate the costly flow simulation. Some of the specific issues related to the immersed-boundary treatment and its parallelization are briefly discussed here. As illustrated in Figure A1b, all nodes on the Cartesian grid are classified into four groups: fluid nodes, solid nodes, ghost nodes, and hybrid nodes. The ghost nodes are located within the solid domain and are immediately adjacent to the fluid-solid interface, and the hybrid nodes are located within the fluid domain and are immediately adjacent to the fluid-solid interface. The solid nodes are simply dummy nodes, while the fluid nodes anchor the standard 2nd-order finite-difference stencil used in the equation discretization. The identification of node types, the set-up of interpolation and extrapolation stencils for the ghost and hybrid nodes, as well as the computations related to updating these nodes during the solution process, are restricted to the subdomain itself. Therefore, the computational overhead associated with the immersed boundary scales very well with the number of subdomains or with the number of processors. Each subdomain is supplemented with two buffer slices on each side to support the interpolation or extrapolation zone for the ghost and hybrid nodes located near the boundary of the subdomain. These slices of data are conveyed through the Message Passing Interface (MPI) in the parallel implementation. Each subdomain also has a full copy of the unstructured surface mesh of the paddling legs. In this work, the bounding box of the whole flow domain is uniformly partitioned into 256 subdomains by employing the two-dimensional domain-decomposition strategy (16 subdomains along both *y* and *z* directions). Thus, a total number of 256 CPU cores is employed.

Independency studies are carried out for mesh and time-step in the case of a single paddling leg with initial swing angle $\alpha_{0}=70^{\circ }$ and swing amplitude $\beta_{0}=20^{\circ }$. For the mesh independency study, three different mesh sizes with Δx=0.30 cm (200×90×160 with about 288 million mesh points), 0.25 cm (228×96×180 with about 394 million points), and 0.20 cm (270×105×210 with about 595 million points) are used in the refined region around the paddling leg. The results of the transient propulsion force ${\bar{F}}_{x}\left(t\right)$ during the first cycle, where t∈[0,0.8] s, are presented in Figure A1c. We can see that the three mesh sizes can capture the major variation characteristics of ${\bar{F}}_{x}\left(t\right)$. Figure A1d presents the results of the time-step independence study. Evidently, ${\bar{F}}_{x}\left(t\right)$ from simulations with time steps Δt=0.0001 s and 0.0002 s agree well with each other, while that from a larger time step Δt=0.0003 s becomes less satisfactory. As a compromise between computation cost and simulation accuracy, Δx=0.25 cm and Δt=0.0002 s are adopted in this work.
